# The Impact of Systematic Review Automation Tools on Methodological Quality and Time Taken to Complete Systematic Review Tasks: Case Study

**DOI:** 10.2196/24418

**Published:** 2021-05-31

**Authors:** Justin Clark, Catherine McFarlane, Gina Cleo, Christiane Ishikawa Ramos, Skye Marshall

**Affiliations:** 1 Institute for Evidence-Based Healthcare Bond University Gold Coast Australia; 2 Bond University Nutrition & Dietetics Research Group Faculty of Health Sciences and Medicine Bond University Gold Coast Australia; 3 Renal Department Sunshine Coast University Hospital Birtinya Australia; 4 Nutrition Programme Federal University of Sao Paulo Sao Paulo Brazil; 5 Department of Science Nutrition Research Australia Sydney Australia

**Keywords:** systematic reviews, automation, technology assessment, methods evaluation, case study, comparison study

## Abstract

**Background:**

Systematic reviews (SRs) are considered the highest level of evidence to answer research questions; however, they are time and resource intensive.

**Objective:**

When comparing SR tasks done manually, using standard methods, versus those same SR tasks done using automated tools, (1) what is the difference in time to complete the SR task and (2) what is the impact on the error rate of the SR task?

**Methods:**

A case study compared specific tasks done during the conduct of an SR on prebiotic, probiotic, and synbiotic supplementation in chronic kidney disease. Two participants (manual team) conducted the SR using current methods, comprising a total of 16 tasks. Another two participants (automation team) conducted the tasks where a systematic review automation (SRA) tool was available, comprising of a total of six tasks. The time taken and error rate of the six tasks that were completed by both teams were compared.

**Results:**

The approximate time for the manual team to produce a draft of the background, methods, and results sections of the SR was 126 hours. For the six tasks in which times were compared, the manual team spent 2493 minutes (42 hours) on the tasks, compared to 708 minutes (12 hours) spent by the automation team. The manual team had a higher error rate in two of the six tasks—regarding *Task 5: Run the systematic search*, the manual team made eight errors versus three errors made by the automation team; regarding *Task 12: Assess the risk of bias*, 25 assessments differed from a reference standard for the manual team compared to 20 differences for the automation team. The manual team had a lower error rate in one of the six tasks—regarding *Task 6: Deduplicate search results*, the manual team removed one unique study and missed zero duplicates versus the automation team who removed two unique studies and missed seven duplicates. Error rates were similar for the two remaining compared tasks—regarding *Task 7: Screen the titles and abstracts* and *Task 9: Screen the full text*, zero relevant studies were excluded by both teams. One task could not be compared between groups—*Task 8: Find the full text*.

**Conclusions:**

For the majority of SR tasks where an SRA tool was used, the time required to complete that task was reduced for novice researchers while methodological quality was maintained.

## Introduction

### Overview

Health care guidelines have reported systematic reviews (SRs) as providing the highest level of evidence to answer research questions [1]. The findings of SRs are favored as they synthesize all published evidence on a topic in a rigorous, reproducible, and transparent way [2]. SRs are used to answer any type of research question, including interventional, diagnostic, prognostic, or etiological [1]; in addition, they are pertinent to many different stakeholders’ groups, from clinicians to researchers to policy makers. However, SRs are time and resource intensive [3] and may be out of date by the time they are published [4]. The time from SR registration to publication has been reported as taking five authors approximately 67 weeks [5], with time frames ranging from 6 months to 2 years [6]. Even rapid reviews, which omit some of the steps of a full SR, have been reported to take 7 to 12 months [7].

To improve time to completion, systematic review automation (SRA) tools have been developed to either fully automate or semi-automate one or more specific tasks involved in conducting an SR. These include searching multiple databases [8], deduplicating search results [9], identifying disagreements between screeners [10,11], and assessing risk of bias (RoB) in randomized controlled trials (RCTs) [12]. In 2015, the International Collaboration for the Automation of Systematic Reviews (ICASR) was formed to enable resource sharing between groups developing SRA tools [13].

However, despite SRA tool availability, the tools have not been translated into practice, primarily due to distrust of the tools [14]. This may be caused by a lack of transparency of machine learning systems and a shortage of studies evaluating the SRA tools [15]. The third ICASR meeting in 2017 identified the need to overcome barriers to SRA uptake [16]. A potential solution is to evaluate SRA tools in a real-world setting, on real SRs, to test their performance. This case study was designed to do that in the health care field of chronic kidney disease.

### Research Questions

When comparing SR tasks done manually, using standard methods, versus those same SR tasks done using SRA tools, (1) what is the difference in time to complete the SR task and (2) what is the impact on the error rate of the SR task?

## Methods

A case study on the methods used to undertake an SR of RCTs delivering a health care intervention was conducted and has been reported according to the STROBE (Strengthening the Reporting of Observational Studies in Epidemiology) statement [17].

### Ethics Approval and Consent to Participate

Ethics approval was not sought; all participants are authors on this manuscript and the SR tasks undertaken were in an SR in which ethical approval was not required.

### Case Study Participants

An expression of interest was sent to the Bond University Faculty of Health Sciences and Medicine, Australia, seeking researchers planning to commence an SR of RCTs. The only group to volunteer had their SR used in this case study. The SR was conducted by a team of four researchers using current Cochrane methodology [2] and reported using PRISMA (Preferred Reporting Items for Systematic Reviews and Meta-Analyses) guidelines [18]. Two of these researchers (CM and CR) were novice researchers completing their first SR under the supervision of two experienced researchers who were not involved in this case study. These two novice researchers (CM and CR) were sampled as the participants on the manual team.

A second expression of interest was sent to the faculty seeking two other researchers not involved in the SR to comprise the automation team. This expression was sent to researchers in the same discipline (ie, nutrition and dietetics) to ensure sufficient knowledge of the SR topic. The only interested candidates (SM and GC) took on the role of the participants on the automation team. As new postdoctoral researchers, they had some experience of being part of an SR team (Table 1).

**Table 1:** Characteristics of study participants’ roles and experience.

Team and participants (initials)		Team role	Research role	Coauthor of completed SRs^a^ (eg, middle author), n	Lead author of completed SRs^a^ (eg, first author), n
**Manual team **					
	CM	Primary	PhD student	0	0
	CR	Secondary	PhD student	0	0
**Automation team **					
	SM	Primary	Postdoctoral researcher	0	3
	GC	Secondary	Postdoctoral researcher	1	0

^a^SR: systematic review; published, accepted for publication, or under review.

### Case Study Systematic Review

The SR used in this study—*Prebiotic, probiotic, and synbiotic supplementation in chronic kidney disease: A systematic review and meta-analysis—*has been published [19]. To complete the SR, four databases were searched, 717 results were deduplicated, 596 titles and abstracts were screened for inclusion, 16 studies were included, and 10 studies were meta-analyzed (Table 2).

**Table 2:** Characteristics of the completed and published systematic reviews (SRs) [19].

SR task	SR task description	Value, n
Run the SR	Databases searched	4
Run the SR	Trial registries searched	2
Deduplicate the search results	Records to be deduplicated	717
Deduplicate the search results	Records left after deduplication	586
Screen the titles and abstracts	Studies to screen	586
Find the full text	Full texts required	40
Screen the full text	Full texts for screening	40
Extract the data	Full-text articles extracted (ie, characteristics of studies and outcomes)	16
Assess the risk of bias	Full-text articles requiring risk-of-bias assessment	16
Write the results	Full-text articles qualitatively synthesized	16
Conduct a meta-analysis	Full-text articles meta-analyzed	10

### The Systematic Review Tasks Conducted in the Study

The manual team conducted the SR tasks required to complete a draft of the background, methods, and results sections of the SR; in total, this comprised 16 SR tasks (Table 3 [8,9,12,20,21]) [22]. The automation team conducted the tasks that had an SRA tool available; this comprised six SR tasks. Where an SR task is normally done by a single investigator, such as deduplicating search results, it was done by a single participant—the primary researcher—on each team. Where an SR task is normally done by two people, such as screening the search results, it was done by two participants—the primary and secondary researchers—on each team.

**Table 3:** List and evaluation criteria of all systematic review (SR) tasks and systematic review automation (SRA) tools used.

SR task No.	SR task	SRA tool used	Evaluation criteria
1	Formulate the question	N/A^a^	N/A
2	Check for similar reviews	N/A	N/A
3	Write the protocol	N/A	N/A
4	Design the systematic search	N/A	N/A
5	Run the systematic search	Polyglot Search Translator [8]	Completed by one participant; the number of different types of errors were counted.
6	Deduplicate the search results	Deduplicator [9]	Completed by one participant; deduplicated EndNote libraries were compared to a deduplicated reference standard data set.
7	Screen the titles and abstracts	SRA-Helper^b^ [20]	Completed by two participants; EndNote libraries of the included and excluded studies were compared. A wrongfully excluded study was considered an error.
8	Find the full text	EndNote, SRA-Helper [20], and SARA^c^ [21]	Completed by one participant; the number of references ordered through the library was compared.
9	Screen the full text	SRA-Helper [20]	Completed by two participants; EndNote libraries of the included and excluded studies were compared.
10	Conduct a citation analysis	N/A	N/A
11	Extract the data	N/A	N/A
12	Assess the risk of bias	RobotReviewer [12]	Completed by two participants; the risk-of-bias assessments were compared to a reference standard created by two experienced systematic reviewers external to the two teams.
13	Synthesize the data	N/A	N/A
14	Rerun the systematic search	N/A	N/A
15	Conduct a meta-analysis	N/A	N/A
16	Write the results	N/A	N/A

^a^N/A: not applicable; this task did not have any relevant SRA tools.

^b^SRA-Helper: Systematic Review Accelerator Helper.

^c^SARA: System for Automatically Requesting Articles.

### The Systematic Review Automation Tools Used in the Study

The decision-making framework used to select the five SRA tools used in this study considered the following: (1) tools that were freely (ie, no cost) available for use, (2) tools that were familiar to the experienced author (JC) in order to aid the participants, (3) availability of help guides, and (4) tools that could be applied to as many tasks as possible.

Polyglot Search Translator [8] was selected to automatically translate search strings between various health databases. Deduplicator was selected to detect duplicate records from the search results, allowing the user to view them and then select which ones to keep and which to discard. The Systematic Review Accelerator Helper (SRA-Helper) was selected to interface with EndNote to enable assignment to groups (ie, screening) using a hot key (eg, the space bar), thereby replacing the normal drag-and-drop method used when screening in EndNote. SRA-Helper was also used to help find the full text by interfacing with EndNote to enable hot keys to conduct a title search for articles in a set of predetermined locations: the Bond University Library catalog, PubMed, and Google Scholar. The System for Automatically Requesting Articles (SARA) was selected to interface directly with the Bond University Library system to request up to 500 full texts at a time with a single click. The fifth and final tool used was the RobotReviewer tool [12]. This tool allows users to upload the PDF of an RCT; it will then provide an RoB assessment in four of the seven domains of the Cochrane Collaboration’s RoB tool [23]: random sequence generation, allocation concealment, blinding of participants and researchers, and blinding of outcome assessment (Table 4).

**Table 4:** Systematic review automation (SRA) tools used in this study.

SR^a^ task No.	SR task	SRA tool used	SRA tool description
5	Run the systematic search	Polyglot Search Translator [8]	This tool translates searches from either a PubMed or Ovid MEDLINE search string into a search string that can be used in multiple other databases.
6	Deduplicate the search results	Deduplicator [9]	This tool allows the uploading of sets of references; it then detects and removes duplicate references.
7	Screen the titles and abstracts	SRA-Helper^b^ [20]	This is an automation script used to move references into groups within EndNote software using a predetermined set of keyboard shortcuts.
8	Find the full text	SRA-Helper [20] and SARA^c^ [21]	SRA-Helper is an automation script used to search predefined locations, such as library websites, PubMed, and Google Scholar. SARA is a tool that allows for the bulk requesting of articles (ie, document delivery) from an institutional library.
9	Screen the full text	SRA-Helper [20]	This is an automation script used to move references into groups within EndNote software using a predetermined set of keyboard shortcuts.
12	Assess the risk of bias (RoB)	RobotReviewer [12]	This is a machine learning system that automatically assesses RoB for four of the seven domains defined by the Cochrane Collaboration’s RoB tool; it also highlights the supporting text for these assessments.

^a^SR: systematic review.

^b^SRA-Helper: Systematic Review Accelerator Helper.

^c^SARA: System for Automatically Requesting Articles.

### Outcomes

The outcomes recorded and compared were (1) the time taken to complete each task (in minutes) and (2) the error rate for each task (count).

### Comparison of Outcomes Between Teams

For the single-participant SR tasks (ie, run the systematic search, deduplicate the search results, and find the full text), the primary manual team participant (CM) was compared to the primary automation team participant (SM). For the dual-participant SR tasks (ie, screen the titles and abstracts, screen the full text, and assess the RoB), the time and errors of the primary and secondary participants on each team were added together.

### Time Taken for the Systematic Review Tasks

The time taken for each SR task was recorded separately for (1) undertaking the SR task and (2) learning about the SR task. Learning about each SR task included discussion with experts, reading help guides, or watching help videos. Time was recorded by each individual participant by noting the time they started work on the SR task and noting the time they finished work on the SR task. The total time spent on each task was calculated by subtracting the start time from the finish time. If a task was split over several work sessions, participants added together the times for each work session for each task to give the total time. Timing was paused if the participants foresaw a delay of 5 minutes or longer. The recording of times by the manual team began at *Task 5: Run the systematic search.* Times reported before this were retrospective estimates made by the participants.

### Measuring the Methodological Quality of Each Systematic Review Task

Methodological quality was measured by the number of errors each team made for each SR task. As most SR tasks, as well as errors made during task performance, differ substantially, so did the way we evaluated each SR task.

### Evaluation of Systematic Review Task 5: Run the Systematic Search

The systematic search was evaluated by counting the number of different types of errors made during the translation process. The errors were determined by a Cochrane information specialist and health librarian (David Honeyman; see Acknowledgments) with over 10 years’ experience. The librarian was blinded as to which team had done the translations. Error criteria are listed in Table S1 in Multimedia Appendix 1.

### Evaluation of Systematic Review Task 6: Deduplicate the Search Results

The deduplicated EndNote libraries were compared to a reference standard data set. This reference standard was created and the comparison made by an experienced information specialist (JC). This reference standard was created blind prior to the results from the manual and automation teams being made available. Any unique studies removed and the number of duplicates missed were recorded as errors.

### Evaluation of Systematic Review Tasks 7 and 9: Screen the Titles and Abstracts and Screen the Full Text

EndNote libraries of the studies after screening and dispute resolution from both teams were compared by an experienced information specialist. An incorrectly excluded study was considered an error. The total number of references that were included and moved to the next task (ie, obtain full text) was also recorded. Any incorrectly excluded studies were sent to the senior author on the published SR, who did not participate in this case study.

### Evaluation of Systematic Review Task 8: Find the Full Text

Both teams ran the EndNote *Find Full Text* feature. Once this was completed and EndNote had automatically found as many full texts as it could, the teams attempted to find the remaining ones. This is when the evaluation between teams started. The number of references that were not found and had to be ordered through the library was the evaluation criterion. However, due to differences in institutional access by participants, the results of this evaluation were not reported.

### Evaluation of Systematic Review Task 12: Assess the Risk of Bias

An RoB reference standard was created by two experienced systematic reviewers: an experienced information specialist and an epidemiologist. RoB assessments were compared to the reference standard by the experienced information specialist, and the number of disagreements with the reference standard were counted. A two-level deviation in the domain rating (eg, a *high* RoB rating instead of a *low* RoB rating) was counted as an error. A single-level deviation in the domain rating (eg, *unclear* RoB instead of *low* RoB) was recorded as a difference of opinion.

## Results

The SR and comparison study began in August 2017. The comparison study was completed at the end of March 2018, while the SR was published in October 2018 [19].

### Time Taken to Conduct Systematic Review Tasks

The approximate time taken for the manual team to produce a draft of the background, methods, and results sections (ie, 16 SR tasks) was 126 hours (Table 5). Approximately 101 hours were spent doing all the tasks, and approximately 25 hours were spent learning about the tasks. For the SR tasks where times were compared (ie, SR *Tasks 5-9* and *12*), the total time taken by the manual team was 41 hours and 33 minutes. The time spent doing the SR tasks was 35 hours and 28 minutes, while the time spent learning about the SR tasks was 6 hours and 5 minutes. The automation team took 11 hours and 48 minutes to complete all the SR tasks. The time spent doing the SR tasks was 10 hours and 30 minutes, while the time spent learning about the SR tasks was 1 hour and 18 minutes (Table 5). The times spent on *Task 12: Assess the RoB* were not equivalent, as the RobotReviewer tool only partially automates the task. It assessed RoB in four of the seven domains, while the manual team assessed RoB in seven of the seven RoB domains.

**Table 5:** Time taken for the manual and automation teams to learn and complete each systematic review (SR) task.

SR task No.	SR task	Total time, hours:minutes			Time doing task, hours:minutes			Time learning task, hours:minutes	
		Manual	Automation	Manual		Automation	Manual		Automation
1	Formulate the question	1:00^a^	N/A^b^	1:00^a^		N/A	0:00		N/A
2	Check for similar reviews	1:00^a^	N/A	1:00^a^		N/A	0:00		N/A
3	Write the protocol	4:00^a^	N/A	4:00^a^		N/A	0:00		N/A
4	Design the systematic search	13:00^a^	N/A	13:00^a^		N/A	0:00		N/A
5	Run the systematic search	6:15	1:20	5:00		0:37	1:15		0:43
6	Deduplicate the search results	2:09	0:36	2:09		0:12	0:00		0:24
7	Screen the titles and abstracts	5:10	3:33	4:40		3:28	0:30		0:05
8	Find the full text	0:50	0:23	0:50		0:18	0:00		0:05
9	Screen the full text	3:29	3:44	3:29		3:44	0:00		0:00
10	Conduct a citation analysis	7:43	N/A	7:43		N/A	0:00		N/A
11	Extract the data	9:42	N/A	9:42		N/A	0:00		N/A
12	Assess the risk of bias	23:40	2:12^c^	19:20		2:11^c^	4:20		0:01^c^
13	Synthesize the data	10:00	N/A	8:00		N/A	2:00		N/A
14	Rerun the systematic search	0:22	N/A	0:22		N/A	0:00		N/A
15	Conduct a meta-analysis	16:00	N/A	10:00		N/A	6:00		N/A
16	Write the results	21:20	N/A	10:40		N/A	10:40		N/A
All tasks	Tasks done by both teams	41:33	11:48	35:28		10:30	6:05		1:18
All tasks	Tasks done by manual team	125:40^a^	N/A	100:55^a^		N/A	24:45^a^		N/A

^a^Approximate time only.

^b^N/A: not applicable; task not done by automation team.

^c^Task partially completed; four of seven domains assessed.

### Quality of the Systematic Review Tasks

The manual team had more errors in *Task 5: Run the systematic search,* with eight types of errors made compared to three by the automation team. Regarding *Task 12: Assess the RoB*, the manual team had a total of 25 differences in opinion from the reference standard compared to only 20 from the automation team. The manual team had fewer errors in *Task 6: Deduplicate the search results* by identifying all duplicates while excluding one unique study, compared to the automation team who missed seven duplicates and removed two unique studies. The teams performed similarly for both SR screening tasks (ie, *Tasks 7* and *9*) (Table 6).

**Table 6:** Quality indicators of each task in the systematic review (SR) process.

SR task No.	SR task	Evaluation criteria	Manual team, n	Automation team, n
5	Run the systematic search	Number of different types of errors made	8	3
6	Deduplicate the search results	Number remaining after deduplication	586	594
6	Deduplicate the search results	Unique studies removed	1	2
6	Deduplicate the search results	Duplicates missed	0	7
7	Screen the titles and abstracts	Studies included	38	38
7	Screen the titles and abstracts	Relevant studies excluded	0	0
8	Find the full text	Full texts ordered from library	—^a^	—
9	Screen the full text	Studies included	30	22
9	Screen the full text	Relevant studies excluded	0	0
12	Assess the risk of bias	Same domain	31	36
12	Assess the risk of bias	Different domain	25	20
12	Assess the risk of bias	Errorss in domain	0	0

^a^Although done by both teams, a difference in institutional library access to journal subscriptions meant these tasks could not be compared.

### Availability of Data and Materials

The data sets used and/or analyzed during this study are available from the corresponding author on reasonable request.

## Discussion

### Principal Findings

To complete a draft of the background, methods, and results of the SR, the manual team took approximately 126 hours. To complete the six SR tasks evaluated in this study, the manual team took approximately 42 hours while the automation team took 12 hours. This equates to potential time savings of 30 hours. Due to the small amount of time taken to learn how to use the SRA tools (ie, 2 hours), the time required to learn how to use SRA tools should not be a barrier to their uptake among novice researchers. Regarding methodological quality of SR tasks done with SRA tools, we found that the error rates between teams was minimal and would not significantly impact on the quality of the SR. The manual team had more errors in two of the SR tasks (*Tasks 5* and *12*) and fewer errors in one SR task (*Task 8*); neither team had errors in two of the SR tasks (*Tasks* *7* and *9*).

The automation team was faster in five of six of the SR tasks compared in this study, where the increased speed of four of the tasks was due to an improvement on a manual process. For instance, to modify search strings, researchers may use the *replace* tool in Microsoft Word to manually change the database syntax, or they may use a drag-and-drop process when screening in EndNote. This replacing of manual, tedious work with an SRA tool is an obvious benefit of automation. The other SR task where the automation team was faster was the RoB assessment. It is important to note that although the time reduction for assessing RoB was substantial in the automation team, this team only assessed four out of the seven domains while the manual team assessed all seven of them. The only SR task where the manual team was faster was *Task 8: Screen the full text*, although the times were similar (209 to 224 minutes; a difference of 7.5 minutes per researcher). This was most likely due to the SR task requiring the reading and comprehension of articles to determine if they were eligible; in this case, the manual team members were more experienced as the SR was on a topic of their expertise. This suggests that for SR tasks where the interpretation or understanding of information plays a major role, there are lessened potential time savings for SRA tools.

The total time difference between the manual team and the automation team was substantial and could be translated to significant cost savings in funded studies. The savings may be attributable to several factors. Due to variations within the novice researchers’ experience (0-3 SRs each), it is likely that the time savings were due in part to participant experience. A lack of blinding and randomization may have contributed bias, where the automation team could have pushed themselves to finish the SR tasks faster than they would under normal circumstances. However, due to the vast time difference between groups and both groups being novice users, it is clear that the SRA tools were the primary contributor to the time savings. This finding has been confirmed in other studies. In an RCT, an SRA tool was found to speed up the translation of search strings across databases by 25%, or 15 minutes, per database [8]. A test of three different screening tools found time savings of 154 to 185 hours for a fully automated approach and 61 to 92 hours for a semi-automated approach [24]. Another test of an automated screening tool on three SRs found a 50% reduction in screening workload in two of the SRs and a 40% reduction in the third [25]. Findings from this study align more with the findings of Wallace et al [25], with time savings between 25% and 50%. Further research is required to replicate and confirm the findings from this study in novice researchers to better understand the estimated time savings produced by SRA tools.

As all participants were novice users of the automation tools, the process to learn a new SRA tool may be comparable to the manual team learning to complete a new SR task. Although the availability of training and support for the SRA tools would have reduced the time spent learning to use them, similar SR training and support is routinely available at universities for standard manual methods.

It currently takes a long time for an SR to go from conception to publication (mean 67.3 weeks) [5]. A recent case study looking at time logs across 12 simulated SRs found the average time to complete an SR (mean 3821 records screened; 20 studies included) was 463 days (66 weeks) and 881 person-hours [26]. Individual tasks required were selecting studies (229 hours, 26%), collecting data (211 hours, 24%), preparing the report (202 hours, 23%), conducting the meta-analysis (149 hours, 17%), and descriptive synthesis (52 hours, 6%) [26]. The SR used in this study [19] was substantially smaller (586 records screened; 16 studies included) and less time was required, but the percentage of time spent on comparable tasks generally aligned: selecting studies consumed 39 person-hours (31%), collecting data consumed 43 hours (35%), preparing the report consumed 26 hours (20%), and conducting the meta-analysis consumed 16 hours (12%).

The total time and person-hours from conception to publication is still substantial for SRs that employ SRA tools [26]. A recent case study found that by focusing on a single SR, using SRA tools, and having experienced reviewers, a medium-sized SR of RCTs (1381 records screened; 8 studies included) could be submitted for publication within 16 calendar days (10 working days; 66 person-hours) from conception [21]. This case study also highlights a significant difference between the findings in a novice versus experienced researcher team already familiar with the tools. However, the topics in the experienced case study and in this case study were different; in addition, further research is required to compare novice and experienced teams’ performance on the same topic for firmer estimates of time and error rates to be obtained. Despite the topic difference, this case study had similarities in that it was a medium-sized review and it only included RCTs.

In the case study completed by the experienced reviewers, approximately 17 hours were required to conduct the six tasks that were completed by the automation team in this study, who took approximately 12 hours. Although the cases are not directly comparable, this suggests that while the experience of the researcher team is relevant, it is likely only a small driver of the time savings.

### Limitations and Strengths of the Study

This study was limited by its case study design, with only a single SR used in the comparison as well as variation in the experience of the novice researchers. The times estimated for *Tasks 1* to *4* of the study have less reliability compared to other steps, which should be considered when interpreting findings. The study was limited by the assessment of each SR task individually, outside of the context of the entire SR, which makes results harder to apply to a full SR done with SRA tools. Additionally, due to the niche nature of the research question, the number of studies identified by the search strategy was small compared to other SRs in health; this may have implications for generalizing to other SRs the overall time required to complete the review. Further, this case study was not registered in a trial or study registry database. A strength of the study is that the time measured was the time that each person engaged in active SR tasks, with breaks excluded from the reported time. Another strength is that the time spent learning about the SR tasks was recorded independently from the time spent doing the tasks. The final strength is that the SR used was a real research project, which means the impact of SRA tools can be shown in a real-world setting.

### Conclusions

For the majority of SR tasks where an SRA tool was used, the time required to complete that task was reduced for novice researchers while methodological quality was maintained. Further research is required to confirm these findings.

## Appendix

Multimedia Appendix 1 Supplementary Table S1: Marking criteria for errors in search string translations.

## Abbreviations

ICASR: International Collaboration for the Automation of Systematic Reviews
PRISMA: Preferred Reporting Items for Systematic Reviews and Meta-Analyses
RCT: randomized controlled trial
RoB: risk of bias
SARA: System for Automatically Requesting Articles
SR: systematic review
SRA: systematic review automation
SRA-Helper: Systematic Review Accelerator Helper
STROBE: Strengthening the Reporting of Observational Studies in Epidemiology
