# Supplementary material for: The Impact of Systematic Review Automation Tools on Methodological Quality and Time Taken to Complete Systematic Review Tasks: Case Study
Source: JMIR Med Educ. 2021 May 31;7(2):e24418. doi: 10.2196/24418 (PMC8204237; doi:10.2196/24418)
Supplement: Multimedia Appendix 1 [file mededu_v7i2e24418_app1.docx]

## Supplementary files

**Supplement 1**

**Table S1: Marking criteria for errors in search string translations**

| **Error type** | **Explanation** |
| --- | --- |
| Incorrect MeSH conversion | Incorrectly converting a Mesh term to, for instance an Emtree |
| Missing subject term | Not including a subject term when translating the search |
| Missing keyword term | Not including a keyword term when converting the search |
| Incorrect field syntax | Incorrectly converting the syntax from one database to another, e.g. using [tiab] in Embase instead of :ti,ab |
| Extra out of place characters inserted | Inserting an extra character during the conversion process, this is most likely a spelling mistake, for instance adding an “s” to the end of a word |
| Incorrect search structure | Doing something that makes the search run as not originally intended, such as missing a bracket. |
| Incorrect phrase translation | Not converting a phrase search correctly, generally means not adding quotations marks around a work for instance using Word to find, instead of “Word to find” |
| Not exploding subject terms | Having the subject search only search for the term, instead of all of the narrower terms that may fall underneath it |
| Chose incorrect fields | Choosing the wrong field, for instance choose an all fields search when it should have been limited to a title and abstract search |
